# Supplementary material for: Estimating the replicability of highly cited clinical research (2004–2018)
Source: PLoS One. 2024 Aug 7;19(8):e0307145. doi: 10.1371/journal.pone.0307145 (PMC11305584; doi:10.1371/journal.pone.0307145)
Supplement: S1 Table — Table shows highly cited studies with their effect measures, the % of sample size they account for in the replication meta-analyses, the published results of these meta-analyses, and those of their reanalyses before and after removing the highly cited study. Out of 16 meta-analyses, we were able to reanalyze 11: EXTEND-IA (12) is an individual patient data meta-analysis, whereas the remaining 3 are network meta-analyses with no direct comparisons. One network meta-analysis (17) replicates two highly cited studies ((13) and (16)), making it 4 network meta-analyses that were not possible to conduct independent replications. One network meta-analysis (18) had direct comparisons in its supplementary materials, making it possible to conduct the independent analysis. Differences between the effect sizes of published and reanalyzed meta-analyses using the same studies occur due to changes in meta-analytical methods and software, but are generally small. (DOCX) [file pone.0307145.s001.docx]

| **Highly cited study** | **Effect Measure** | **% of Sample Size** | **Published effect size [95% CI]** | **Effect size with highly cited study [95% CI]** | **Effect size without highly cited study [95% CI]** |
| --- | --- | --- | --- | --- | --- |
| Topalian et al. 2012 (1) | ORR | 8.2 | 0.26 [0.21; 0.31] | 0.24 [0.20; 0.30] | 0.25 [0.19; 0.31] |
| Brahmer et al. 2012 (2) | ORR | 5.5 | 0.27 [0.21; 0.33] | 0.25 [0.19; 0.32] | 0.26 [0.20; 0.33] |
| SYNTAX (3) | OR | 16.7 | 1.42 [1.27; 1.59] | 1.43 [1.27; 1.59] | 1.43 [1.27; 1.61] |
| ACCORD (4) | RR | 17.6 | 0.92 [0.85; 1.00] | 0.92 [0.85; 0.99] | 0.92 [0.85; 0.99] |
| ECASS III (5) | OR | 11.9 | 1.29 [1.16; 1.43] | 1.28 [1.13; 1.45] | 1.37 [1.07; 1.74] |
| MR CLEAN (6) | RR | 17.2 | 1.37 [1.14; 1.64] | 1.47 [1.20; 1.82] | 1.44 [1.15; 1.82] |
| ESCAPE (7) | RR | 10.9 | 1.37 [1.14; 1.64] | 1.47 [1.20; 1.82] | 1.43 [1.14; 1.80] |
| ERSPC (8) | IRR | 24.3 | 0.96 [0.85; 1.08] | 0.98 [0.86; 1.12] | 1.02 [0.93; 1.12] |
| CATIE (9) | HR | 28.6 | 0.68 [0.56; 0.83] | 0.68 [0.55; 0.84] | 0.69 [0.52; 0.91] |
| HERA (10) | HR | 23 | 0.65 [0.55; 0.75] | 0.66 [0.58; 0.76] | 0.62 [0.56; 0.68] |
| EURTAC (11) | HR | 32 | 0.23 [0.17; 0.30] | 0.30 [0.17; 0.53] | 0.26 [0.10; 0.67] |
| EXTEND-IA (12) | OR | 5.60 | 4.04 [2.75; 5.93] | - | - |
| SHARP (13) | HR | 42.1 | 0.69 [0.60; 0.79] | - | - |
| TAXUS-IV (14) | RR | 13.52 | 0.66 [0.59; 0.74] | - | - |
| PROFILE 1007 (15) | HR | 38.68 | 0.46 [0.39; 0.54] | - | - |
| Cheng et al. 2009 (16) | HR | 27.29 | 0.69 [0.54; 0.87] | - | - |
