# Supplementary material for: Estimating the replicability of highly cited clinical research (2004–2018)
Source: PLoS One. 2024 Aug 7;19(8):e0307145. doi: 10.1371/journal.pone.0307145 (PMC11305584; doi:10.1371/journal.pone.0307145)
Supplement: S2 Table — Rates consider only independent primary studies (i.e. RCTs, phase II trials) and meta-analyses that do not include the highly cited studies. Meta-analyses that could not be reanalyzed were excluded from the analysis. Otherwise, results are displayed in the same way as in Table 4. (DOCX) [file pone.0307145.s002.docx]

**Table S2**

| **Criterion** | **Total** | **Replicated** | **% Replicated [95% CI]** |
| --- | --- | --- | --- |
| Statistical significance | 10 | 8 | 80% [49, 94] |
| 95% CI overlap | 19 | 16 | 84% [62, 94] |
| 95% CI overlap and statistical significance | 19 | 15 | 79% [57, 91] |
| Statistical significance including negative studies | 12 | 8 | 67% [41, 94] |
| Replication estimate within highly cited study’s 95% CI | 19 | 11 | 58% [36, 77] |
| Highly cited study estimate within replication 95% CI | 19 | 9 | 47% [27, 68] |
