# Supplementary material for: Estimating the replicability of highly cited clinical research (2004–2018)
Source: PLoS One. 2024 Aug 7;19(8):e0307145. doi: 10.1371/journal.pone.0307145 (PMC11305584; doi:10.1371/journal.pone.0307145)
Supplement: S3 Table — Rates consider only independent primary studies (i.e. RCTs, phase II trials) and meta-analyses that do not include the highly cited studies. Meta-analyses that could not be reanalyzed were excluded from the analysis. Otherwise, results are displayed in the same way as in Table 5. (DOCX) [file pone.0307145.s003.docx]

**Table S3**

| **Analysis** | **All** | **p-value** | **Phase 1 trials** | **RCTs** |
| --- | --- | --- | --- | --- |
| Main analysis | 0.99 [0.83 - 1.19] | 0.93 | 1.02 [0.66 - 1.57] | 0.98 [0.79 - 1.20] |
| Publication order | 1.06 [0.89 - 1.27] | 0.48 | 1.02 [0.66 - 1.57] | 1.09 [0.89 - 1.33] |
| Effect coining | 1.04 [0.87 - 1.23] | 0.67 | 1.17 [0.80 - 1.71] | 0.96 [0.79 - 1.18] |
| Publication order + coining | 1.11 [0.94 - 1.31] | 0.21 | 1.17 [0.80 - 1.71] | 1.07 [0.88 - 1.31] |
