# Supplementary material for: Estimating the replicability of highly cited clinical research (2004–2018)
Source: PLoS One. 2024 Aug 7;19(8):e0307145. doi: 10.1371/journal.pone.0307145 (PMC11305584; doi:10.1371/journal.pone.0307145)
Supplement: S4 Table — Rates consider only independent primary studies (i.e., RCTs, phase II trials) and meta-analyses that do not include the highly cited studies. Meta-analyses that could not be reanalyzed for this purpose were excluded from the analysis. Otherwise, results are displayed in the same way as in Table 6. Sample size for each group varies according to the specific criteria: statistical significance: 8 replicated, 2 contradicted; confidence interval overlap: 16 replicated, 3 contradicted; both criteria: 15 replicated, 4 contradicted. (DOCX) [file pone.0307145.s004.docx]

**Table S4.**

| **Predictor** | **Criteria** | **Median (IQR) Replicated** | **Median (IQR) Contradicted** | **p-value** |
| --- | --- | --- | --- | --- |
|  | Significance (p < 0.05) (independent) | 308 [243 - 406] | 441 [338 - 543] | 0.71 |
| Citations/year of the highly cited study | CI overlap [independent] | 337 [247 - 414] | 494 [368 - 570] | 0.36 |
|  | Significance [p < 0.05] & CI overlap [independent] | 346 [269 - 434] | 368 [241 - 532] | 0.81 |
|  | Significance [p < 0.05] [independent] | 2x10^-5^ [4x10^-6^ - 2x10^-3^] | 0.02 [8x10^-3^ - 0.02] | 0.53 |
| p-value of the highly cited study | CI overlap [independent] | 5x10^-4^ [5x10^-5^ - 0.03] | 1x10^-5^ [1x10^-5^ - 1x10^-5^] | 0.83 |
|  | Significance [p < 0.05] & CI overlap [independent] | 3x10^-4^ [5x10^-6^ - 0.03] | 0.02 [8x10^-3^ - 0.02] | 0.91 |
|  | Significance [p < 0.05] [independent] | 580 [287 – 1066] | 91152 [45729 – 136576] | 0.71 |
| Sample size of the highly cited study | CI overlap [independent] | 498 [194 – 1066] | 207 [131 – 256] | 0.21 |
|  | Significance [p < 0.05] & CI overlap [independent] | 495 [187 – 760] | 256 [169 – 45729] | 0.81 |
| **Predictor** | **Criteria** | **# replicated by study design** | **# not replicated by study design** | **p-value** |
| Highly cited study design | CI overlap [independent] | Phase 1 trial: 5/7 RCT: 11/12 | Phase 1 trial: 2/7 RCT: 1/12 | 0.52 |
|  | Significance [p < 0.05] & CI overlap [independent] | Phase 1 trial: 5/7 RCT: 10/12 | Phase 1 trial: 2/7 RCT: 2/12 | 0.60 |
|  | Significance [p < 0.05] [independent] | Pharmacological: 4/5  Other: 4/5 | Pharmacological: 1/5  Other: 1/5 | 1.00 |
| Type of intervention | CI overlap [independent] | Pharmacological: 10/13  Other: 6/6 | Pharmacological: 3/13  Other: 0/6 | 0.52 |
|  | Significance [p < 0.05] & CI overlap [independent] | Pharmacological: 10/13  Other: 5/6 | Pharmacological: 3/13  Other: 1/6 | 1.00 |

**References**

1. Topalian SL, Hodi FS, Brahmer JR, Gettinger SN, Smith DC, McDermott DF, et al. Safety, Activity, and Immune Correlates of Anti–PD-1 Antibody in Cancer. N Engl J Med. 28 de junho de 2012;366(26):2443–54.

2. Brahmer JR, Tykodi SS, Chow LQM, Hwu WJ, Topalian SL, Hwu P, et al. Safety and Activity of Anti–PD-L1 Antibody in Patients with Advanced Cancer. N Engl J Med. 28 de junho de 2012;366(26):2455–65.

3. Serruys PW, Morice MC, Kappetein AP, Colombo A, Holmes DR, Mack MJ, et al. Percutaneous Coronary Intervention versus Coronary-Artery Bypass Grafting for Severe Coronary Artery Disease. N Engl J Med. 5 de março de 2009;360(10):961–72.

4. The Action to Control Cardiovascular Risk in Diabetes Study Group TA to CCR in DSG, Gerstein H, Miller M, Byington R, Goff Jr D, Bigger T, et al. Effects of Intensive Glucose Lowering in Type 2 Diabetes. N Engl J Med. 12 de junho de 2008;358(24):2545–59.

5. Hacke W, Kaste M, Bluhmki E, Brozman M, Dávalos A, Guidetti D, et al. Thrombolysis with Alteplase 3 to 4.5 Hours after Acute Ischemic Stroke. N Engl J Med. 25 de setembro de 2008;359(13):1317–29.

6. Berkhemer OA, Fransen PSS, Beumer D, van den Berg LA, Lingsma HF, Yoo AJ, et al. A Randomized Trial of Intraarterial Treatment for Acute Ischemic Stroke. N Engl J Med. janeiro de 2015;372(1):11–20.

7. Goyal M, Demchuk AM, Menon BK, Eesa M, Rempel JL, Thornton J, et al. Randomized Assessment of Rapid Endovascular Treatment of Ischemic Stroke. N Engl J Med. 12 de março de 2015;372(11):1019–30.

8. Schröder FH, Hugosson J, Roobol MJ, Tammela TLJ, Ciatto S, Nelen V, et al. Screening and Prostate-Cancer Mortality in a Randomized European Study. N Engl J Med. 26 de março de 2009;360(13):1320–8.

9. Lieberman JA, Stroup TS, McEvoy JP, Swartz MS, Rosenheck RA, Perkins DO, et al. Effectiveness of Antipsychotic Drugs in Patients with Chronic Schizophrenia. N Engl J Med. 22 de setembro de 2005;353(12):1209–23.

10. Piccart-Gebhart MJ, Procter M, Leyland-Jones B, Goldhirsch A, Untch M, Smith I, et al. Trastuzumab after Adjuvant Chemotherapy in HER2-Positive Breast Cancer. N Engl J Med. 20 de outubro de 2005;353(16):1659–72.

11. Rosell R, Carcereny E, Gervais R, Vergnenegre A, Massuti B, Felip E, et al. Erlotinib versus standard chemotherapy as first-line treatment for European patients with advanced EGFR mutation-positive non-small-cell lung cancer (EURTAC): a multicentre, open-label, randomised phase 3 trial. Lancet Oncol. 1o de março de 2012;13(3):239–46.

12. Campbell BCV, Mitchell PJ, Kleinig TJ, Dewey HM, Churilov L, Yassi N, et al. Endovascular Therapy for Ischemic Stroke with Perfusion-Imaging Selection. N Engl J Med. 12 de março de 2015;372(11):1009–18.

13. Llovet JM, Ricci S, Mazzaferro V, Hilgard P, Gane E, Blanc JF, et al. Sorafenib in Advanced Hepatocellular Carcinoma. N Engl J Med. 24 de julho de 2008;359(4):378–90.

14. Stone GW, Ellis SG, Cox DA, Hermiller J, O’Shaughnessy C, Mann JT, et al. A Polymer-Based, Paclitaxel-Eluting Stent in Patients with Coronary Artery Disease. N Engl J Med. 15 de janeiro de 2004;350(3):221–31.

15. Shaw AT, Kim DW, Nakagawa K, Seto T, Crinó L, Ahn MJ, et al. Crizotinib versus Chemotherapy in Advanced ALK-Positive Lung Cancer. N Engl J Med. 20 de junho de 2013;368(25):2385–94.

16. Cheng AL, Kang YK, Chen Z, Tsao CJ, Qin S, Kim JS, et al. Efficacy and safety of sorafenib in patients in the Asia-Pacific region with advanced hepatocellular carcinoma: a phase III randomised, double-blind, placebo-controlled trial. Lancet Oncol. 1o de janeiro de 2009;10(1):25–34.

17. Niu M, Hong D, Ma TC, Chen XW, Han JH, Sun J, et al. Short-term and long-term efficacy of 7 targeted therapies for the treatment of advanced hepatocellular carcinoma: a network meta-analysis: Efficacy of 7 targeted therapies for AHCC. Medicine (Baltimore). dezembro de 2016;95(49):e5591.

18. Zhao Y, Liu J, Cai X, Pan Z, Liu J, Yin W, et al. Efficacy and safety of first line treatments for patients with advanced epidermal growth factor receptor mutated, non-small cell lung cancer: systematic review and network meta-analysis. BMJ. 7 de outubro de 2019;367:l5460.
