# Supplementary figures and images for: Estimating the replicability of highly cited clinical research (2004–2018)
Source: PLoS One. 2024 Aug 7;19(8):e0307145. doi: 10.1371/journal.pone.0307145 (PMC11305584; doi:10.1371/journal.pone.0307145)

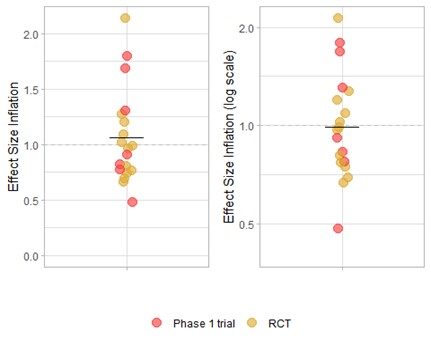

Supplement: S1 Fig — Rates consider only independent primary studies [i.e. RCTs, phase II trials] and meta-analyses that do not include the highly cited studies. Meta-analyses that could not be reanalyzed for this purpose were excluded from the analysis. Otherwise, results are displayed in the same way as in Fig 3. Lines indicate the mean of the plotted values (which in the left panel differs from that on S3 Table, calculated on the basis of log-transformed values). (TIFF) [file pone.0307145.s005.tiff]
